# Sinister seafood: bacteraemia secondary to non-O1/O139 *Vibrio cholerae* infection

**DOI:** 10.1099/jmmcr.0.005103

**Published:** 2017-07-05

**Authors:** Maxime J. Billick, Philip W. Lam, Isaac I. Bogoch

**Affiliations:** ^1^​ Faculty of Medicine, McGill University, Montreal, QC, Canada; ^2^​ Department of Medicine, Division of Infectious Diseases, University of Toronto, Toronto, ON, Canada

**Keywords:** *Vibrio*, cholera, bacteraemia, *Vibrio cholerae*, gastroenteritis

## Abstract

**Introduction.** *Vibrio* species are curved, motile Gram-negative bacilli found in estuarine and marine environments, and are known to cause to gastroenteritis, skin and soft tissue infections, and septicaemia. While not responsible for cholera epidemics, non-O1/O139 *Vibrio cholerae* (NOVC) is increasingly reported as a cause of gastroenteritis.

**Case presentation.** A 66-year-old man presented to an emergency department with a 1 week history of epigastric pain, emesis and fever. Blood cultures drawn on admission initially demonstrated Gram-negative bacilli, and ultimately grew NOVC, which was later confirmed by matrix-assisted laser desorption ionization-time of flight MS. Subsequent history revealed that the patient had eaten fish and seafood prior to falling ill. He was treated with intravenous ceftriaxone and oral doxycycline while admitted, and oral ciprofloxacin and doxycycline upon discharge. His bacteraemia was believed to be secondary to altered gut anatomy from prior surgery and proton-pump inhibitor use.

**Conclusion.** Risk factors for NOVC bacteraemia include cirrhosis, immunosuppression and other forms of liver disease. Cases are often linked to a history of seafood ingestion when water temperatures rise, enabling *Vibrio* species to proliferate. While the optimal management of NOVC bacteraemia is unclear, a combination of a third-generation cephalosporin with a tetracycline has been suggested. Physicians should maintain a high index of suspicion for this pathogen when evaluating ill patients with a history of liver disease and seafood ingestion.

## Abbreviation

NOVC, non-O1/O139 Vibrio cholerae.

## Introduction

*Vibrio* species are curved, motile Gram-negative bacilli found in estuarine and marine environments [1]. The three most common *Vibrio* species implicated in human illness are *Vibrio parahaemolyticus*, *Vibrio vulnificus* and *Vibrio cholerae*.

The United States Centers for Disease Control and Prevention estimate that 80 000 cases of vibriosis occur yearly in the USA, resulting in 500 hospitalizations and 100 deaths [2]. Vibriosis cases are associated with syndromes of gastroenteritis, skin and soft tissue infections, and septicaemia [2].

*V. cholerae* is classified based on the O-antigen polysaccharide type, with over 200 serogroups described. Historically, only toxigenic serogroups O1 and O139 have been associated with widespread cholera epidemics [3]. Although other serogroups may cause smaller outbreaks, they are collectively termed non-O1/O139 *Vibrio cholerae* (NOVC). Severe illness with NOVC bacteraemia is a rare event; however, there have been numerous documented case reports of severe wound infections, gastroenteritis and sepsis [1]. Due to its rare presentation and potential for poor clinical outcomes, it is important to recognize NOVC bacteraemia in the clinical setting.

## Case report

A 66-year-old man presented to an emergency room with a 1 week history of epigastric pain, emesis and fever. His medical history was significant for a benign pancreatic tumour causing biliary obstruction managed with a biliary stent, cholecystectomy, choledochojejunostomy and roux-en-Y reconstruction years before his presentation. His medications included pancrealipase, rabeprazole and insulin. With regards to social history, the patient had emigrated from Hong Kong 30 years previously and had no recent travel history or sick contacts. He was febrile (38.2 °C) but otherwise haemodynamically stable, and the remainder of his exam was only notable for mild epigastric and right upper quadrant tenderness, audible bowel sounds and no peritoneal signs.

## Investigations

Laboratory findings demonstrated a mild leukocytosis (13.3×10^9^ cells l^−1^ white blood cell count) and mildly elevated liver enzymes (aspartate aminotransferase, 71 U l^−1^; alanine aminotransferase, 62 U l^−1^) with a normal blood glucose level of 6.6 mmol l^−1^. The remainder of his initial laboratory findings were normal. Two sets of blood cultures drawn on admission demonstrated Gram-negative bacilli on Gram stain, and were positive for NOVC via culture and confirmed by matrix-assisted laser desorption ionization-time of flight (MALDI-TOF) MS. The bacteria were plated on blood agar, chocolate agar, MacConkey agar and fastidious anaerobic agar, and grew on all four plates within 24 h. The organism was found to be susceptible to ampicillin, azithromycin, trimethoprim*/*sulfamethoxazole and tetracycline. A subsequent history that was taken revealed that the patient consumed home-prepared seafood hot pot and fish at a restaurant 3 and 5 days before symptom onset, respectively; thus, indicating a potential infectious source.

## Treatment

The patient was treated empirically with oral doxycycline (100 mg, twice daily) and intravenous ceftriaxone (1 g, daily) prior to *V. cholerae* speciation. He rapidly defervesced and was discharged home on a 7 day course of oral ciprofloxacin (500 mg) and doxycycline (100 mg), both taken twice daily.

## Outcome and follow-up

The patient was at his baseline level of function with a normalized complete blood count and liver enzyme testing at a follow up appointment 1 month after discharge.

## Discussion

*Vibrio* species are curved, motile Gram-negative bacilli found in estuarine and marine environments [1]. The three most common *Vibrio* species implicated in human illness are *V. parahaemolyticus*, *V. vulnificus* and *V. cholerae*. The United States Centers for Disease Control and Prevention estimates 80 000 cases of vibriosis occur yearly in the USA, resulting in 500 hospitalizations and 100 deaths [2]. *Vibrio* may cause gastroenteritis if ingested, aggressive wound infections from percutaneous injuries and septicaemia [2].

Although severe illness with NOVC bacteraemia is uncommon, case reports of wound infections, gastroenteritis and sepsis have been documented [1]. Risk factors for NOVC bacteraemia include cirrhosis, immunosuppression and other forms of liver disease [1, 2]. Additionally, cases are often linked to a history of seafood ingestion [1] in summer months when water temperatures rise, resulting in prime conditions for *Vibrio* species to proliferate [2]. We suspect the history of seafood ingestion, combined with altered gastrointestinal anatomy and proton pump inhibitor use, contributed to the pathogenesis of infection in our case. [4]. No family members, close contacts or others who shared meals with our patient had clinical illness, and we did not screen them for infection.

The optimal management of NOVC bacteraemia is unclear. A combination of a third-generation cephalosporin with a tetracycline have been suggested, based on *in vivo* evidence demonstrating synergy and the theoretical benefits of protein synthesis inhibition and reduced toxin production [1]. Furthermore, empiric dual antibiotic coverage may be warranted in light of increasing antimicrobial resistance.

Although NOVC infections have not been linked to epidemics, these bacteria are associated with severe wound infections, gastroenteritis and sepsis in vulnerable hosts. As sea surface temperatures continue to rise globally [5], there is the risk of increased vibrio proliferation. Physicians should maintain a high index of suspicion for this pathogen when evaluating ill patients with a history of liver disease, seafood ingestion or marine exposure.
